# O8.4. THE EFFECT OF EARLY MEDICATION DISCONTINUATION ON LONG-TERM CLINICAL OUTCOME IN FIRST EPISODE PSYCHOSIS

**DOI:** 10.1093/schbul/sby015.240

**Published:** 2018-04-01

**Authors:** Eric Chen, Christy Hui, W C Chang, Sherry Chan, Edwin Lee, William Honer

**Affiliations:** 1 University of Hong Kong; 2 University of British Columbia

## Abstract

**Background:**

Clinical decision to dis/continue antipsychotics in patients remitted from first-episode psychosis is important. Existing short-term evidence suggests that patients who discontinued antipsychotics had more relapses. Data on long-term outcomes are lacking; with only one open-label study suggesting better long-term recovery outcome in patients who had early medication discontinuation. We examined the long-term effect of early medication discontinuation in year 2 following first-episode remission for patients with no residual psychotic symptoms.

**Methods:**

We followed-up 178 first-episode psychosis patients who participated in a 1-year randomized controlled trial (RCT) on medication discontinuation. Patients were randomized into receiving either a medication maintenance group or a placebo discontinuation group. After the RCT, all patients received usual psychiatric care. Poor long-term clinical outcome was defined as a composite of persistent psychotic symptoms, a requirement for clozapine, or suicide.

**Results:**

There were no differences between patients who were included (n=142) and excluded (n=36) from the study with regard to their baseline demographics, clinical and functioning. At 10 years, more patients in the early discontinuation group (35/89, 39%) had poor clinical outcome than patients in the maintenance group (19/89, 21%) (P<0.01). Relapse during the RCT has partly mediated the significant relationship between early medication discontinuation and poor outcome at 10-year.

**Discussion:**

Whether to discontinue medication following successful treatment of first episode psychosis is a difficult clinical decision. In first episode psychosis with a full initial response to antipsychotic treatment, continued need for medication is important for the first three years after starting treatment, to prevent relapse, and decrease the risk for a poor long-term outcome.

